# Biomechanical and morphological evaluation of aortic wall changes related to the cross-clamping method in different surgical techniques

**DOI:** 10.1590/1677-5449.200204

**Published:** 2021-12-01

**Authors:** Pedro Pereira Tenório, Rodrigo Mendes, José Honório de Almeida Palma da Fonseca

**Affiliations:** 1 Universidade Federal do Vale do São Francisco – UNIVASF, Colegiado de Medicina, Campus Paulo Afonso, BA, Brasil.; 2 Universidade Federal de São Paulo – UNIFESP, Escola Paulista de Medicina, São Paulo, SP, Brasil.; 3 Hospital das Clínica da Faculdade de Medicina da Universidade de São Paulo – HCFMUSP, Instituto do Coração – InCor, São Paulo, SP, Brasil.

Dear Editor,

The objective of the authors of “Acute aortic wall injury caused by aortic cross-clamping: morphological and biomechanical study of the aorta in a swine model of three aortic surgery approaches”^1^ was to assess changes to the aorta wall related to the clamping method used in different aortic surgery techniques. Their biomechanical study detected significant differences between the different surgical techniques studied. Specimens in the EV (endovascular) group proved to be more resistant to load than those from the other groups.

Whenever a vessel is handled, the possibility exists of plaque rupture, intimal injury, and formation of thrombi during and after placement of the hemostatic clamps or endovascular balloon. These injuries are predominantly dependent on the variables time and pressure applied, which lead to distortion of the intima and disarrangement of the tunica media, leading to weakening of the aorta wall, as previous studies have demonstrated.^2^^,^^3^

However, in this study, the histology of the aorta was unchanged. No changes were observed in collagen or elastic fibers and no cellular changes were observed either – the nuclei of the smooth muscle cells (SMCs) were intact. The Abstract of the article mentions that morphometric techniques were employed, but the methodology was not described. It was also unclear how many areas were selected in each field, in order to ensure standardization.

The reduction in vascular resistance may have been caused both by destruction of the extracellular matrix - an event that leads to activation of the SMC apoptosis process^4^ - and by buildup of mucoid material, which is of strongly anionic character, probably because of the presence of proteoglycans (PGs). Bearing in mind that the distensibility of the aorta is not exclusively because of collagen and elastin, but is also due to the presence of PGs, and that all of these can affect the resistance of the aorta to traction,^5^ analysis of PGs would be indispensable. Such an analysis could have been conducted by staining with alcian blue and toluidine blue to mark sulphated and carboxylated structures, respectively,^6^ followed by morphometric quantification.^7^

Thus, considering that the elastic capacity of cardiovascular tissues is directly proportional to their biomechanical behavior, it would be expected that some histological changes would have occurred along group lines and would have resulted in findings detected by the biomechanical experiments.
