# Supplementary material for: Biomechanical and morphological evaluation of aortic wall changes related to the cross-clamping method in different surgical techniques
Source: J Vasc Bras. 2021 Dec 1;20:e20200204. doi: 10.1590/1677-5449.200204 (PMC8719455; doi:10.1590/1677-5449.200204)
Supplement: Translation

# Translation: Avaliações biomecânica e morfológica das alterações da parede aórtica relacionadas ao método de interrupção de fluxo em diferentes técnicas cirúrgicas

**Authors:** Pedro Pereira Tenório, Rodrigo Mendes, José Honório de Almeida Palma da Fonseca

**Affiliations:** 1 Universidade Federal do Vale do São Francisco – UNIVASF, Colegiado de Medicina, Campus Paulo Afonso, BA, Brasil.; 2 Universidade Federal de São Paulo – UNIFESP, Escola Paulista de Medicina, São Paulo, SP, Brasil.; 3 Hospital das Clínica da Faculdade de Medicina da Universidade de São Paulo – HCFMUSP, Instituto do Coração – InCor, São Paulo, SP, Brasil.

Caro Editor,

No artigo intitulado “Lesão aguda da parede arterial provocada pelo método de interrupção temporária de fluxo em diferentes vias de cirurgia aórtica: estudo morfológico e biomecânico da aorta de porcos”^1^, os autores objetivaram avaliar as alterações da parede aórtica relacionadas ao método de interrupção de fluxo em diferentes técnicas de cirurgia da aorta. No estudo biomecânico, houve diferenças significativas entre as distintas técnicas cirúrgicas. O grupo EV (endovascular) mostrou-se mais resistente à carga quando comparado aos demais grupos.

Sempre que um vaso é manipulado, há possibilidade de rotura de placas, lesão intimal e formação de trombos durante e após o posicionamento das pinças hemostáticas ou do balão endovascular. Essa lesão é dependente, sobretudo, das variáveis tempo e pressão empregada, que levam a uma distorção da íntima e a um desarranjo da camada média, conduzindo a um enfraquecimento da parede aórtica, como estudos prévios já demonstraram^2^^,^^3^.

Porém, no estudo, a histologia da aorta apresentou-se preservada. Não foram observadas alterações nas fibras colágenas e elásticas nem alterações celulares – o núcleo das células musculares lisas (CMLs) estavam preservados. No resumo do artigo, foi mencionado o emprego da técnica morfométrica, mas sua metodologia não foi descrita. Além disso, não ficou claro quantas áreas foram selecionadas em cada campo, a fim de garantir uma padronização.

A diminuição da resistência vascular poderia ser justificada tanto pela destruição da matriz extracelular, evento que conduz à ativação do processo apoptótico das CMLs^4^, quanto pelo acúmulo de material mucoide, que apresenta caráter fortemente aniônico, provavelmente devido à presença de proteoglicanos (PGs). Tendo em vista que a distensibilidade da aorta se deve não apenas ao colágeno e à elastina, mas também aos PGs, e que todos podem afetar a resistência da aorta à tração^5^, seria imprescindível a análise dos PGs. Essa análise poderia ter sido realizada mediante o emprego do azul de alcian e azul de toluidina para marcação de compostos sulfatados e carboxilados, respectivamente^6^, seguida da quantificação morfométrica^7^.

Logo, tendo em vista que a capacidade elástica dos tecidos cardiovasculares é diretamente proporcional ao seu comportamento biomecânico, era de se esperar uma alteração histológica concordante com os grupos que apresentaram alterações nos ensaios biomecânicos.

# Translation

**Authors:** Rodrigo Gibin Jaldin

Prezado Editor,

Os pontos considerados são absolutamente pertinentes e foram abordados na discussão dos resultados, particularmente o papel da matriz extracelular (proteoglicanos), o qual não foi avaliado no estudo. Os autores consideram que esses são os pontos fracos do estudo, o que também consta na discussão do artigo, e deixam como horizonte pesquisas futuras com o objetivo de eventualmente mensurar os componentes da matriz extracelular.
