# Supplementary material for: Biomechanical and morphological evaluation of aortic wall changes related to the cross-clamping method in different surgical techniques
Source: J Vasc Bras. 2021 Dec 1;20:e20200204. doi: 10.1590/1677-5449.200204 (PMC8719455; doi:10.1590/1677-5449.200204)
Supplement: Related texts

# Reply

**Authors:** Rodrigo Gibin Jaldin

Dear Editor,

The points made are entirely pertinent and were covered in the discussion dos results, particularly the role of the extracellular matrix (proteoglycans), which was not investigated in the study. The authors consider these to be the study’s weak points, which is also mentioned in the discussion in the article, and indicate them as avenues for future research with the objective of measuring extracellular matrix components.
